# BaltDC: purification, characterization and infrared spectroscopy of an antiplatelet DC protein isolated from *Bothrops alternatus* snake venom

**DOI:** 10.1186/s40409-017-0126-7

**Published:** 2017-07-28

**Authors:** Mariana Santos Matias, Bruna Barbosa de Sousa, Déborah Fernanda da Cunha Pereira, Edigar Henrique Vaz Dias, Carla Cristine Neves Mamede, Mayara Ribeiro de Queiroz, Anielle Christine Almeida Silva, Noelio Oliveira Dantas, Andreimar Martins Soares, Júnia de Oliveira Costa, Fábio de Oliveira

**Affiliations:** 1 Postgraduate Program in Genetics and Biochemistry, Institute of Genetics and Biochemistry, Federal University of Uberlândia (UFU), Uberlândia, MG Brazil; 2 Institute of Agricultural Sciences, Federal University of Uberlândia (UFU), Monte Carmelo, MG Brazil; 3 Institute of Biomedical Sciences, Federal University of Uberlândia (UFU), Uberlândia, MG Brazil; 4 Institute of Physics, Federal University of Uberlândia (UFU), Uberlândia, MG Brazil; 5 Center for the Study of Biomolecules Applied to Health (CEBio), Oswaldo Cruz Foundation (Fiocruz – Rondônia) and Health Group, Federal University of Rondônia (UNIR), Porto Velho, RO Brazil; 6 University Center São Lucas (UniSL), Porto Velho, RO Brazil; 7 Federal Institute of Education, Science and Technology of Triângulo Mineiro (IFTM), Campus Ituiutaba, Ituiutaba, MG Brazil; 8 National Institute of Science and Technology in Nanobiopharmaceutics (N-Biofar), Belo Horizonte, MG Brazil

**Keywords:** Snake venom, *Bothrops alternatus*, DC protein, Platelet aggregation

## Abstract

**Background:**

Snake venoms are a complex mixture of proteins, organic and inorganic compounds. Some of these proteins, enzymatic or non-enzymatic ones, are able to interact with platelet receptors, causing hemostatic disorders. The possible therapeutic potential of toxins with antiplatelet properties may arouse interest in the pharmacological areas. The present study aimed to purify and characterize an antiplatelet DC protein from *Bothrops alternatus* snake venom.

**Methods:**

The protein, called BaltDC (DC protein from *B. alternatus* snake venom), was purified by a combination of ion-exchange chromatography on DEAE-Sephacel column and gel filtration on Sephadex G-75. The molecular mass was estimated by polyacrylamide gel electrophoresis in the presence of sodium dodecyl sulfate (SDS-PAGE). The amino acid sequence of the N-terminal region was carried out by Edman degradation method. Platelet aggregation assays were performed in human platelet-rich plasma (PRP). Infrared (IR) spectroscopy was used in order to elucidate the interactions between BaltDC and platelet membrane.

**Results:**

BaltDC ran as a single protein band on SDS-PAGE and showed apparent molecular mass of 32 kDa under reducing or non-reducing conditions. The N-terminal region of the purified protein revealed the amino acid sequence IISPPVCGNELLEVGEECDCGTPENCQNECCDA, which showed identity with other snake venom metalloproteinases (SVMPs). BaltDC was devoid of proteolytic, hemorrhagic, defibrinating or coagulant activities, but it showed a specific inhibitory effect on platelet aggregation induced by ristocetin and epinephrine in PRP. IR analysis spectra strongly suggests that PO_3_^2−^ groups, present in BaltDC, form hydrogen bonds with the PO_2_^**−**^ groups present in the non-lipid portion of the membrane platelets.

**Conclusions:**

BaltDC may be of medical interest since it was able to inhibit platelet aggregation.

## Background

Isolation and characterization of pharmacologically active compounds present in snake venoms have been the focus of numerous scientific research studies. Approximately 90% of snake venom dry weight is constituted of protein components, including peptides and enzymes. The non-protein fraction of the venom is composed by low-mass organic molecules, such as carbohydrates, lipids, free amino acids and inorganic compounds including calcium, phosphorus, magnesium, potassium, sodium and zinc [1, 2].

Snake venoms are rich sources of metalloproteinases, a group of enzymes that are the primary factors responsible for hemorrhage and may also interfere with the hemostatic system [3–6]. Snake venom metalloproteinases (SVMPs) have been classified into three classes, from PI to PIII, according to their multi-domain structure [7]. The PIII SVMPs are the largest among all the classes of metalloproteinases. They are composed of an N-terminal metalloproteinase domain, a disintegrin-like domain (D) and a Cys-rich C-terminus (C). PIII SVMPs may undergo proteolytic processing, releasing the catalytic domain (metalloproteinases) [7]. D and C domains are linked by disulfide bonds, so they are released as a unique molecule (DC protein). This molecule is able to interact with integrins present on the cell surface and may trigger numerous cellular processes such as platelet aggregation; angiogenesis; metastasis; tumor growth; adhesion, migration and proliferation of cells [8–12].

In this work, we describe the purification, characterization and IR spectra of an antiplatelet DC protein, called BaltDC, isolated from *B. alternatus* snake venom.

## Methods

### *B. alternatus* snake venom

Desiccated *B. alternatus* snake venom was purchased from Bioagents Serpentarium (Brazil). This serpentarium is registered in the Brazilian Institute of Environment and Renewable Natural Resources (IBAMA – n. 471,301). The crude venom was dried in a vacuum desiccator at room temperature immediately after milking and then stored at −20 °C.

### Animals

Swiss male mice (20–25 g) were provided by the Center of Animal Facilities and Animal Experimentation (CEBEA) of the Federal University of Uberlândia (Uberlândia, MG, Brazil). The animals were maintained under conditions of controlled temperature (22 ± 2 °C) and 12-h light/dark cycles with free access to food and water. The experimental protocol was approved by the Committee for Ethics in Animal Experimentation of the Federal University of Uberlândia (CEUA/UFU, protocol number 108/12).

### Human blood

Human blood was obtained by means of donation from volunteers. The criteria for selection of donors were: be in good state of health, have 18 to 65 years old, weighting at least 50 kg, no use of any medication that interferes with hemostasis, no use of illicit drugs and no alcohol consumption for at least 24 h before donation. The experiments were carried out according to the current guidelines for research with humans established by the Committee for Ethics in Human of the Federal University of Uberlândia (CEP/UFU – protocol number 1.627.982/2016).

### Isolation of BaltDC

*B. alternatus* crude venom (300 mg) was dissolved in 2.0 mL of 0.05 M ammonium bicarbonate buffer (pH 7.8) and applied to a DEAE-Sephacel column (2.5 × 20 cm). The samples were eluted using a linear gradient (0.05–1.0 M) of the same buffer. The ninth peak was pooled, lyophilized and applied to a Sephadex G-75 column (1.0 × 100 cm) previously equilibrated with 0.05 M ammonium bicarbonate buffer (pH 7.8). All peaks were monitored by measuring absorbance at 280 nm on a spectrophotometer BioSpec-Mini (Shimadzu Biotech, Japan) at a flow rate of 20 mL/h and fractions of 3.0 mL/tube were collected. The purified protein was named BaltDC. To confirm the degree of purity, BaltDC was submitted to reverse-phase Source 15RPC ST column (4.6 × 100 mm) using the ÄKTApurifier™ HPLC system. The column was equilibrated with 0.1% trifluoroacetic acid (solvent A) and eluted with a linear concentration gradient from 0 to 100% of 70% acetonitrile, 0.1% trifluoroacetic acid (solvent B) at a flow rate of 0.3 mL/min. Absorbance was monitored at 280 nm.

### Estimation of protein concentration

Protein concentration was determined by the method previously described by Bradford [13], using bovine serum albumin as standard.

### Electrophoretic analysis

Polyacrylamide gel electrophoresis in the presence of sodium dodecyl sulfate (SDS-PAGE) was performed as described by Laemmli [14] using 14% (*w*/*v*) gels. Electrophoresis was carried out at 20 mA/gel in Tris-glycine buffer (pH 8.3) containing 0.01% SDS. The molecular mass standard proteins used were phosphorylase b (97 kDa), bovine serum albumin (66 kDa), ovalbumin (45 kDa), carbonic anhydrase (30 kDa), soybean trypsin inhibitor (20.1 kDa) and α-lactalbumin (14.4 kDa). Gels were stained with Coomassie blue R-250, 0.2% (*w*/*v*).

### N-terminal sequencing

A PPSQ-33A (Shimadzu) automated sequencer was used for the N-terminal sequencing according to the methodology described by Rodrigues et al. [15]. The identity of the primary sequence of BaltDC, compared with other proteins, was evaluated using BLAST (http://blast.ncbi.nlm.nih.gov/Blast.cgi).

### Platelet aggregation assay

Platelet aggregation assays were performed in PRP and measured using the automated Aggregometer 4 channels (AggRAMTM version 1.1, Helena Laboratories, USA) as described by Queiroz et al. [16]. Human blood, collected in the presence of sodium citrate (3.2%), was centrifuged at 100×*g* for 12 min at room temperature to obtain PRP. Platelet-poor plasma (PPP) was obtained from the residue by centrifugation of citrated blood at 1000×g for 15 min. Assays were carried out using 200 μL of PRP maintained at 37 °C under continuous stirring in siliconized glass cuvettes. Aggregation was triggered with collagen (10 μg/mL), ADP (20 μM), ristocetin (1.5 mg/mL) or epinephrine (300 μM) with BaltDC (20, 40 and 80 μg). One hundred percent (100%) aggregation was expressed as the percentage absorbance relative to PPP aggregation. Control experiments were performed using only platelet agonists. All experiments were carried out in triplicate.

### Infrared spectra

IR spectra of the samples were recorded at room temperature using a Shimadzu Fourier Transform IR (FT-IR) spectrophotometer (Vertex 70, Bruker Optik) in the spectral range 440 to 4000 cm^−1^ via a total attenuated reflectance element coupled (ATR) with resolution of 2 cm^−1^.

## Results and discussion

**Fig. 1:**
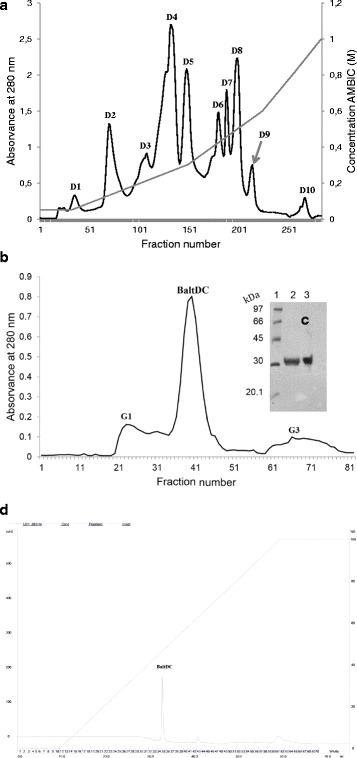
Purification of BaltDC.**(a)** Anion-exchange chromatography of *B. alternatus* crude venom on a DEAE-Sephacel column (2.5 × 20 cm) equilibrated with 0.05 M ammonium bicarbonate (pH 7.8) and eluted with a convex concentration gradient of the same buffer (0.05–1 M). **(b)** Gel filtration on Sephadex G-75 column (1.0 × 100 cm): ninth peak was applied to the column and eluted with 0.05 M ammonium bicarbonate. Fractions of 3.0 mL/tube were collected and the absorbance was read at 280 nm. **(c)** SDS-PAGE: Lane 1 – standard proteins; lane 2 – BaltDC under non-reducing conditions; lane 3 – BaltDC under reducing conditions. The molecular mass standard proteins used were phosphorylase b (97 kDa), bovine serum albumin (66 kDa), ovalbumin (45 kDa), carbonic anhydrase (30 kDa) and soybean trypsin inhibitor (20.1 kDa). Gels were stained with Coomassie blue R-250, 0.2%. **(d)** Reverse-phase HPLC on a Source 15RPC ST column (4.6 × 100 mm) equilibrated with 0.1% trifluoroacetic acid (TFA) and eluted with a linear concentration gradient from 0 to 100% of solution B (70% acetonitrile in 0.1% TFA)

In Brazil, the *B. alternatus* snake, popularly known as *urutu cruzeiro* or *cruzeira*, is found in swamps, marshes and other humid local. It is also commonly found in sugarcane plantations [17]. In this work, we described the purification (chromatographic steps), electrophoretic profile, N-terminal sequence and IR spectra of an antiplatelet DC protein from this snake venom. The protein was isolated by only two steps of purification (ion-exchange and gel filtration chromatography). *B. alternatus* crude venom (300 mg) was applied on a DEAE-Sephacel column and produced ten main protein peaks (Fig. 1a). The ninth peak was further fractionated by size exclusion chromatography (Sephadex G- 75), resulting in three main peaks (Fig. 1b). The second peak of this chromatography, which was named BaltDC, was devoid of proteolytic, hemorrhagic, defibrinating or coagulant activities (data not shown), but it is able to interfere on platelet aggregation.

**Fig. 2:**
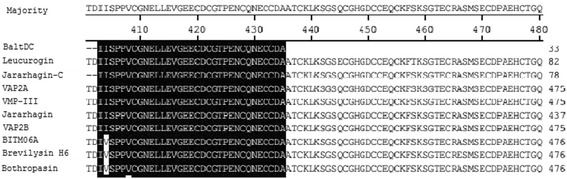
Sequence alignment of BaltDC and members of the PIII SVMPs: jararhagin-C (AAB30855.1), leucurogin (P0DJ87.1), VAP2A (A4PBQ9.1), VMP-III (C9E1R8.1), jararhagin (P30431.1), VAP2B (Q90282.1), BITM06A (Q8QG88.1), brevilysin-H6 (P0C7B0.2) and bothropasin (O93523.2). The conserved residues are shown in black. The alignment and figure were generated and evaluated using BLAST (http://blast.ncbi.nlm.nih.gov/Blast.cgi)

Electrophoretic (SDS-PAGE) analysis under denaturing and reducing conditions indicated that BaltDC had an apparent molecular mass of 32 kDa (Fig. 1c). The high degree of purity of BaltDC was confirmed by reverse-phase HPLC chromatography on a Source 15RPC ST column, revealing a unique major peak (Fig. 1d). Comparison of the N-terminal sequence of BaltDC (IISPPVCGNELLEVGEECDCGTPENCQNECCDA) showed similarity with other PIII SVMPs from *Bothrops* genus. The N-terminal of BaltDC is similar to the middle of others metalloproteinases skipping the catalytic domain such as leucurogin, jararhagin-C, VAP2A, VMP-III, jararhagin, bothropasin and others (Fig. 2) [18–23].

The PIII SVMPs are high molecular mass proteins that have a proteinase domain followed by disintegrin-like (D) and cysteine-rich (C) domains. Jia et al. [24] and Serrano et al. [25] reported the hypothesis that the DC domain contributes to the proteolytic specificity of PIII SVMPs, since it is targeted to bind to critical protein substrates. On the other hand, studies with synthetic peptides, such as the one by Pinto et al. [26], suggest that the C domain of the jararhagin binds to vWF. According to Fox and Serrano [27], some proteinases from snake venom may generate biologically active intact polypeptides of approximately 30 kDa that correspond to the DC protein, which may be released from their precursor forms by proteolytic processing.

Brevilysin-H6, bothropasin, leucurolysin B and jararhagin are PIII SVMPs composed of a metalloproteinase domain associated with a DC domain, which may be released from auto-proteolytic events [5, 22, 23, 28]. BaltDC is similar to jararhagin C (28 kDa), acucetin (30 kDa) and alternagin-C (29 kDa), which are DC proteins released through auto-proteolysis from *B. jararaca, A. acutus* and *B. alternatus*, respectively [12, 19, 29]. As jararhagin C, acutin and alternagin-C, BaltDC has no enzymatic activity. These results associated with the N-terminal sequence leads us to believe that the BaltDC may be a fragment of class PIII SVMPs, in which the DC domain was processed from proteinase domain, as proposed by Fox and Serrano [7].

**Fig. 3:**
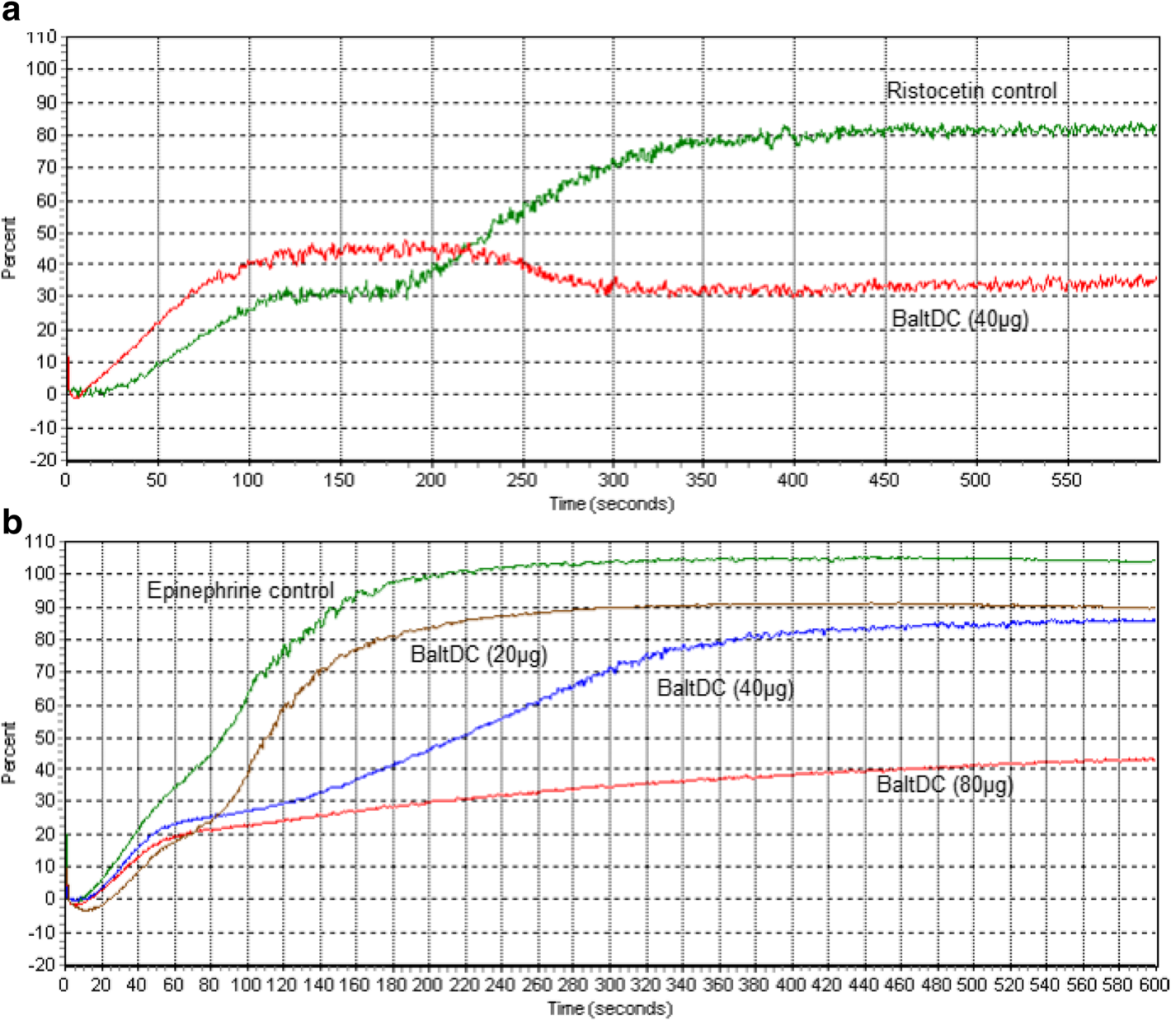
Effect of BaltDC on **(a)** ristocetin and **(b)** epinephrine-induced platelet aggregation. PRP was preincubated with BaltDC for 30 min at 37 °C before adding agonists. Platelet aggregation was recorded for 10 min in an AggRAM platelet aggregation system with four-channel laser optics (Helena Laboratories, EUA). Results were expressed as an increase in light transmission. Control experiments were performed using only platelet agonists

DC proteins are known to bind to different platelet integrins that mediate platelet aggregation. A number of DC proteins have been used in the studies of modulators of platelet adhesion receptors and their ligands [12, 30, 31]. In this work, we characterized the interference of BaltDC on platelet aggregation using collagen, ADP, epinephrine and ristocetin as agonist. Our results showed that 40 μg and 80 μg of BaltDC was able to inhibit approximately 60% of platelet aggregation induced by ristocetin and epinephrine, respectively (Fig. 3a and b). On the other hand, BaltDC had little or no effect on platelet aggregation induced by collagen or ADP (data not shown). These results suggest that BaltDC inhibits platelet aggregation by a possible common pathway for ristocetin and epinephrine. Another PIII SVMPs, such as acurhagin, purified from the venom of *Agkistrodon acutus*, also inhibits the ristocetin-induced platelet aggregation by hydrolyzing the vWF [32]. Our results strongly suggest that BaltDC inhibits platelet aggregation by preventing the binding of vWF and epinephrine to their respective receptors since it does not have a catalytic effect. Probably, BaltDC does not interact with the αIIbβ3, αVβ3 or α2β1x integrins since it does not inhibit the aggregation induced by ADP or collagen. However, more data are needed to elucidate the mechanism of action of BaltDC.

Here, we also show some results that may contribute to the understanding of a type of interaction that occurs between BaltDC and the platelet membrane. In order to elucidate these interactions, we used the IR spectroscopy. This methodology is not invasive and it has been used as an extremely useful tool for the investigation of interactions between lipids and proteins and other biological and biomedical studies [33–38].

**Fig. 4:**
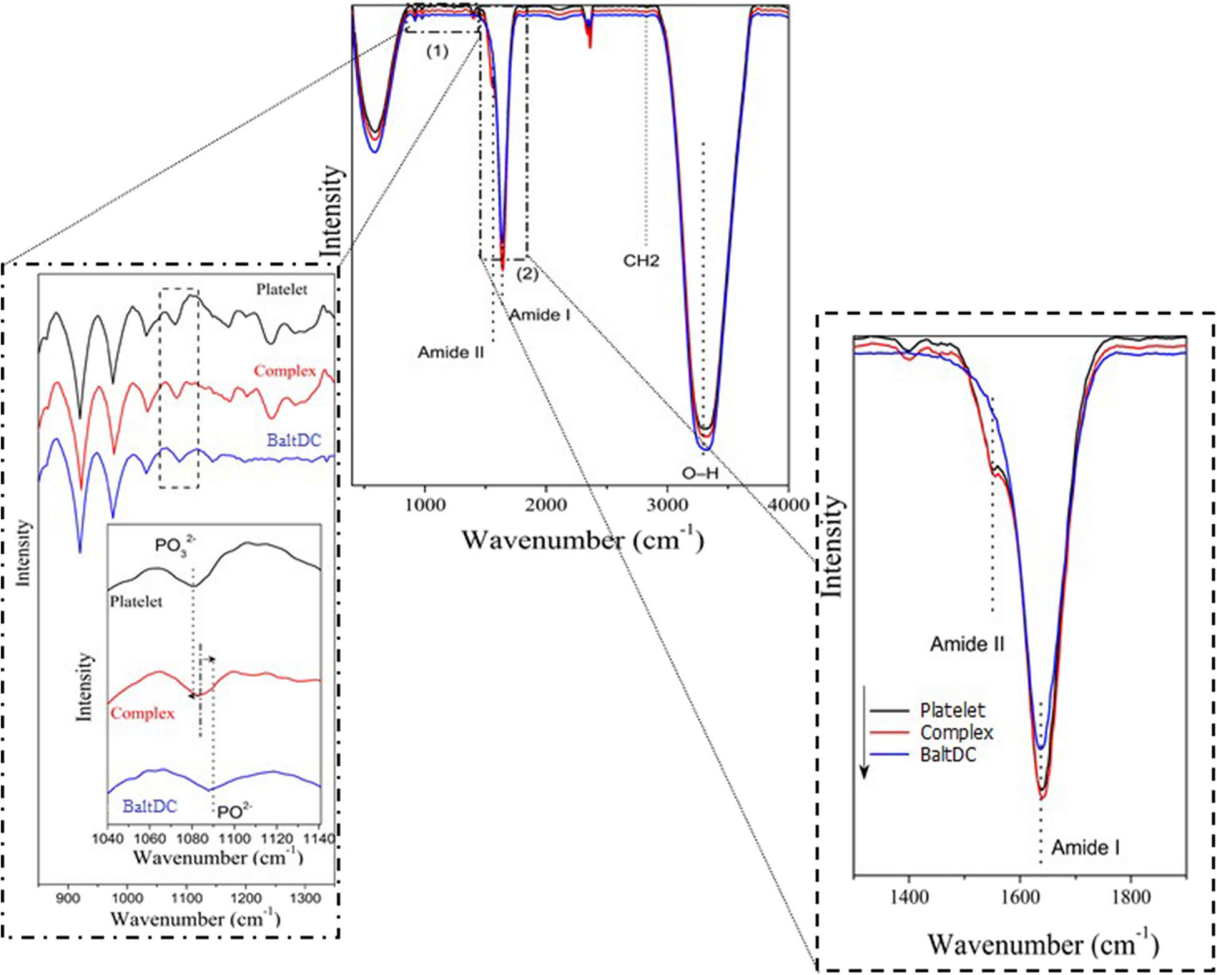
FT-IR Spectra of BaltDC, platelet and complex (BaltDC/platelet). Zoom 1:850–1350 cm^−1^. Zoom 2: 1300–1900 cm^−1^. IR spectra were recorded by a total attenuated reflectance element coupled (ATR) with resolution of 2 cm^−1^

Fig. 4 shows the FT-IR spectra of BaltDC and platelet alone and when they are complexed. In the region 850–1350 cm^−1^ (zoom 1), we observed a variety of characteristic IR group frequencies similar in all spectra. This same figure shows that the spectrum of BaltDC has a band at 1087 cm^−1^ while the spectrum of platelet has a band at 1080 cm^−1^, which are characteristics of PO_2_^−^ and PO_3_^2−^ symmetric stretching vibrations, respectively [39, 40]. Interestingly, the spectrum of BaltDC/platelet complex showed a band at 1083 cm^−1^, which presented a change of the frequencies relative to the spectra of BaltDC and platelet, when they are separated. These results support the hypothesis that the PO_3_^2−^ groups, present in BaltDC, make hydrogen bonds with the PO_2_^**−**^ groups present on the non-lipid portion of the membrane platelets [41–43]. We believe that the PO_2_^**−**^ groups are part of protein complexes, which are buried in the lipid bilayer since these groups are not commonly found in the plasma membrane. In addition, no changes were observed in the characteristic spectral bands of phosphate groups generally found in the membrane phospholipids [44].

In the region 1300–1900 cm^−1^ (zoom 2), we observed in both FT-IR spectra, BaltDC and platelets, a band at 1640 cm^−1^, located in amide I region, which indicates α-helical conformation [38, 45]. The spectrum of protein /platelet complex shows that the bands corresponding to amide I and II regions remained unchanged. These results suggest that the interaction between BaltDC and platelets causes no conformational changes in membrane, prevailing the α-helix structure [46]. These findings support the hypothesis that BaltDC does not act catalytically and therefore it could act as an antagonist of the ristocetin and epinephrine receptors.

## Conclusions

We presented the purification, characterization and IR spectrum of BaltDC, a DC protein (32 kDa) originated from autolysis of a PIII SVMPs from *B. alternatus* snake venom. This protein was able to inhibit platelet aggregation induced by ristocetin and epinephrine and, therefore, it may be of medical interest as a novel therapeutic antiplatelet agent.

## Abbreviations

BaltDC: DC protein isolated from *Bothrops alternatus* snake venom
DC protein: D (disintegrin-like) and C (Cys-rich C-terminus) domains are released as a unique molecule
HPLC: High performance liquid chromatography
IR: Infrared
PPP: Platelet-poor plasma
PRP: Platelet-rich plasma
SDS-PAGE: Polyacrylamide gel electrophoresis in the presence of sodium dodecyl sulfate
SVMP: Snake venom metalloproteinase
